# Hemorrhagic Stroke in Relapsing Polychondritis: A Rare Complication of a Rare Disease

**DOI:** 10.1155/2020/7464503

**Published:** 2020-02-21

**Authors:** Benjamin Chaucer, Augustus Demanes, Abriella Stone, Venkat Kakollu

**Affiliations:** University of Illinois College of Medicine at Peoria, Peoria, Illinois, USA

## Abstract

**Background:**

Relapsing Polychondritis is a rare rheumatologic condition with multisystem involvement. Common presenting symptoms are auricular and nasal chondritis. Common complications include hearing loss and cardiac involvement. An extremely rare complication is neurological involvement. *Case report*. We present a case of relapsing polychondritis resulting in stroke and discuss the current literature on this condition.

**Conclusion:**

To our knowledge, only 6 previous documented cases of stroke secondary to relapsing polychondritis exist in the current literature. This case represents a rare but important complication of relapsing polychondritis.

## 1. Introduction

Relapsing Polychondritis is a rare rheumatologic condition. It is a multisystem disease which affects tissues high in proteoglycans throughout the body. Common presenting symptoms are auricular and nasal chondritis. Complications vary widely and range from hearing loss to cardiac involvement. An extremely rare complication is vasculitis and neurologic involvement. We present a unique case of relapsing polychondritis resulting in stroke and discuss the current literature on this condition.

## 2. Case Report

The patient was a 41-year-old female who presented to the emergency room with chest pain of a one-day duration with odynophagia and auricular chondritis of a 12-week duration. She denied cough, shortness of breath, nausea, vomiting, or heart burn. Odynophagia and ear pain had been present for three months and had progressed until she was unable to tolerate any oral intake. Her ear pain was constant and characterized by ache in her lobes and the cartilage of her ears. During her interview, she was holding both ears and speaking in a hoarse voice while taking pauses to swallow painfully. Electrocardiogram showed sinus rhythm with no ST segment changes. Troponin was 0.02. Ventilation perfusion scan was normal. Despite her negative cardiac and pulmonary embolism work up, she endorsed continued chest and throat pain. On physical exam, the patient had a 4/6 systolic murmur at the left 2^nd^ intercostal space. Gross inspection of the oropharynx showed no erythema without any clear evidence of trauma. Chart review showed that the patient had a degenerative aortic valve in her adolescence requiring aortic valve replacement, with subsequent replacement again 13 years prior to presentation with a porcine valve. Transesophageal echo was performed, which showed severe thickening of the leaflets with a mobile density, consistent with a vegetation attached to the aortic valve. Blood cultures were drawn, and the patient was started on vancomycin 2 gm IV QD with Piperacillin/Tazobactam 4.5 gm QID IV for empiric treatment of infective endocarditis. She remained afebrile with all blood cultures negative for bacteremia. Cardiology was consulted and found the vegetation to be noninfectious degeneration of her aortic valve. Antibiotic treatment was subsequently stopped. Gastroenterology was consulted and performed esophagogastroduodenoscopy (EGD) which showed normal appearing esophageal mucosa, with an inflamed, erythematous, ulcerated vallecula. Inspection of the stomach showed diffuse gastric mucosal erythema with diffuse gastritis. Biopsy results from duodenum and stomach were negative for both malignancy and *H. pylori* infection. She was treated with viscous lidocaine, morphine, and Toradol with no pain relief. ENT specialists performed flexible laryngoscopy which showed discolored mucus coating the vallecula and epiglottis. Biopsy results showed ulcerated squamous mucosa with dense mixed and focal granulomatous inflammation, granulation tissue, and destruction of underlying cartilage (see Supplementary image 1). ANCA, MPO, ANA, and PR3 were drawn and were all negative. Given the patient's positive histological findings, bilateral auricular chondritis, and degeneration of heart valve, she was diagnosed with relapsing polychondritis. Treatment was initiated with solumedrol 125 mg QD for a three-day course. On day 2, she developed an acute bilateral, retro-orbital headache, with photophobia and altered mental status. In-house stroke alert was called, and CT head was performed which showed basilar subarachnoid hemorrhage. The patient was transferred to the neurosurgical intensive care unit (ICU) where she was intubated, and external ventricular drain was placed to relieve intracranial pressure. After 2 days in the ICU, she was extubated without difficulty. Rehabilitation therapy was initiated in anticipation of discharge. After appropriate rehabilitation, the patient was discharged home.

## 3. Discussion

Relapsing polychondritis (RP) is a rare rhuemetologic condition that affects multiple organ systems. Its annual incidence is estimated at 3.5 cases per million per year with a median age of 50 [1]. Any tissue can be affected (see Table 1); however, involvement predominates in cartilaginous tissue.

The most common manifestations of relapsing polychondritis are inflammation of the ear involving the pinna and sparing the lobe (89%), nasal cartilage (61%), ocular inflammation (59%), and arthritis (79%). Reversible conductive and sensorineural hearing loss is also common in 40% of patients [2]. Nasal involvement can result in clinical findings of saddle nose deformity, which is seen in approximately 28% of cases. An extremely rare complication is stroke. While aortic involvement is documented, vasculitis resulting in stroke is rare. Patients often complain of auricular and nasal chondritis, hearing loss, and peripheral neuropathy [3]. RP is an inflammatory condition highly associated with HLA-DR4 [4]. Diagnostic criteria were described by McAdam et al. [3, 5, 6]. Diagnosis of relapsing polychondritis can be confirmed if three out of the following six criteria are met: recurrent chondritis of both auricles, nonerosive polyarthritis, chondritis of nasal cartilages, inflammation of ocular structures, involvement of laryngeal or tracheal cartilage, and cochlear or vestibular damage. These criteria were initially proposed by McAdam et al. and later slightly modified by Damiani and Levine. Damiani and Levine suggested a diagnosis could be made when one or more of the abovementioned features were present along with a positive biopsy or when two or more separate sites of cartilage inflammation were present that responded to glucocorticoids or dapsone or when three or more features were present [7]. In our patient, diagnosis was made by positive clinical findings of recurrent chondritis of both auricles, nonerosive inflammatory polyarthritis, laryngeal chondritis, and sensorineural hearing loss with vertigo along with positive biopsy findings. A study performed by Kings College in London analyzing 106 cases of RP showed the standardized mortality ratio of 2.16% with the leading cause of death being respiratory and cardiac disease and cancer [8]. Treatment of active relapsing polychondritis is 40–60 mg/d of prednisone which is tapered once the disease is controlled. Patients with severe organ-threatening disease, who fail to respond effectively to prednisone, could be considered for immunosuppressive drugs such as azathioprine, cyclosporine, methotrexate, or cyclophosphamide. Aortic valve dysfunction and aneurysm have been well documented in RP. When grouped together, cardiac complications represent the second most common cause of death in RP, with cardiac involvement found in 10–25% of cases [4]. Early cardiac involvement as was seen in our patient is rare. An exceedingly rare complication of RP is stroke. Our literature search showed only 6 cases of stroke secondary to RP in the modern literature [9–14]. Central nervous system and peripheral nervous system involvement only account for 3% of complications seen in RP [4]. Antiglutamate receptor epsilon2 (NR2B) autoantibodies have been found in RP patients with limbic encephalitis [15]. Whether this antibody plays a role in the pathogenesis of stroke in RP is unknown. The exact etiology of RP is not clearly defined nor is its role in stroke. Further research is needed to elucidate the relationship between relapsing polychondritis and stroke.

## Figures and Tables

**Table 1:** Complications of RP by site involvement.

	(%)
Pinna with sparing of the lobe	89
Arthritis	79
Nasal cartilage	61
Ocular inflammation	59
Reversible conductive and sensorineural hearing loss	40
Saddle nose deformity	29

## Supplementary Materials

Supplementary Materials Biopsy results showed ulcerated squamous mucosa with dense mixed and focal granulomatous inflammation.

## References

[1] Kent P. D., Michet C. J., Luthra H. S. (2004). Relapsing polychondritis. *Current Opinion in Rheumatology*.

[2] Luthra H. S., Stone J. H. (2009). Relapsing polychondritis. *A Clinician’s Pearls and Myths in Rheumatology*.

[3] McAdam L. P., O’Hanlan M. A., Bluestone R., Pearson C. M. (1976). Relapsing polychondritis: prospective study of 23 patients and a review of the literature. *Medicine (Baltimore*.

[4] Zeuner M., Straub R. H., Rauh G., Albert E. D., Scholmerich J., Lang B. (1997). Relapsing polychondritis: clinical and immunogenetic analysis of 62 patients. *The Journal of Rheumatology*.

[5] Michet C. J., McKenna C. H., Luthra H. S., O’Fallon W. M. (1986). Relapsing polychondritis: survival and predictive role of early disease manifestations. *Ann Intern Med*.

[6] Damiani J. M., Levine H. L. (1979). Relapsing polychondritis-report of ten cases. *Laryngoscope*.

[7] Harrisons (1998). *Harrison’s Principles of Internal Medicine*.

[8] Hazra N., Dregan A., Charlton J., Gulliford M. C., D’Cruz D. P. (2015). Incidence and mortality of relapsing polychondritis in the UK: a population-based cohort study. *Rheumatology*.

[9] Kilic Coban E., Gez S., Kara B., Soysal A. (2015). A rare complication of a rare disease; stroke due to relapsing polychondritis. *Ideggyógyászati Szemle*.

[10] Hsu K., Wu Y., Lyu R., Tang L. (2006). Aseptic meningitis and ischemic stroke in relapsing polychondritis. *Clinical Rheumatology*.

[11] Bouton R., Capon A. (1994). Stroke as initial manifestation of relapsing polychondritis. *The Italian Journal of Neurological Sciences*.

[12] Hull R. G., Morgan S. H. (1984). The nervous system and relapsing polychondritis. *Neurology*.

[13] Karapanayiotides T., Kouskouras K., Ioannidis P., Polychroniadou E., Grigoriadis N., Karacostas D. (2014). Internal carotid artery floating thrombus in relapsing polychondritis. *Journal of Neuroimaging*.

[14] Coban E. K., Gez S., Kara B., Soysal A. (2015). A rare complication of a rare disease; stroke due to relapsing polychondritis. *Ideggyógyászati Szemle*.

[15] Kashihara K., Kawada S., Takahashi Y. (2009). Autoantibodies to glutamate receptor GluRepsilon2 in a patient with limbic encephalitis associated with relapsing polychondritis. *Journal of the Neurological Sciences*.

