# Supplementary figures and images for: Hemorrhagic Stroke in Relapsing Polychondritis: A Rare Complication of a Rare Disease
Source: Case Rep Rheumatol. 2020 Feb 21;2020:7464503. doi: 10.1155/2020/7464503 (PMC7056989; doi:10.1155/2020/7464503)

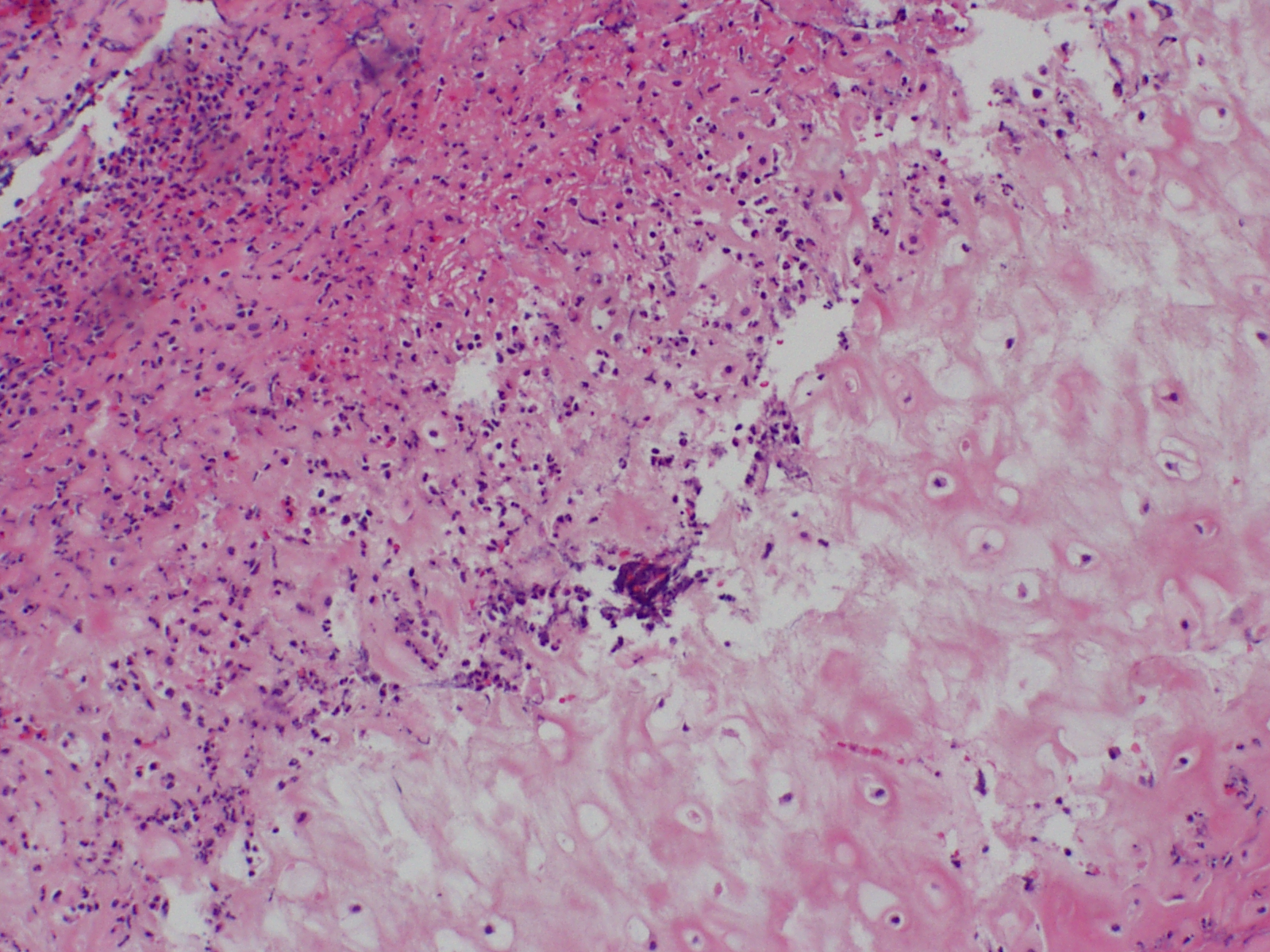

Supplement: Supplementary Materials — Biopsy results showed ulcerated squamous mucosa with dense mixed and focal granulomatous inflammation. [file 7464503.f1.tif]
